# Supplementary material for: Tracking Excess Deaths (TRACKED) – an interactive online tool to monitor excess deaths associated with the COVID-19 pandemic in the United Kingdom
Source: Wellcome Open Res. 2020 Nov 20;5:168. Originally published 2020 Jul 16. [Version 2] doi: 10.12688/wellcomeopenres.16058.2 (PMC11411238; doi:10.12688/wellcomeopenres.16058.2)
Supplement: Peer review history

# Reviewer report: Reviewer response for version 2

**DOI:** 10.21956/wellcomeopenres.18112.r95091

**Published:** 2024-10-08

**Authors:** Gashirai Mbizvo

**Affiliations:** 1 University of Liverpool Institute of Systems Molecular and Integrative Biology, Liverpool, England, UK

Cathie and colleagues have undertaken a nice study helping to track excess mortality during a pandemic using multiple publically available sources, ascribing what proportion of the deaths are related to COVID itself and what proportion are not (or not at least directly). This will allow for planning of services to cater for wider morbidity and mortality outside of COVID. The methods are appropriate to the aims of the study. The tool will be helpful for other researchers to use. In particular, the data sources used can be quite a challenge to navigate and analyse, so it is nice to have a tool that pools the data and does that for one. 

 I have only one minor comment - I wonder if they might wish to integrate the timing of the various national vaccine implementations so we can see if these had any visible potential associations with the trends seen in both COVID and non-COVID excess deaths. This may help with some of the potential explainability of trends seen. It would also be, for example, interesting to see if there is a decline in non-covid excess deaths in trend with vaccination as it would suggest vaccines have a wider impact on herd protection beyond direct primary COVID prevention.

Is the work clearly and accurately presented and does it cite the current literature?

Yes

If applicable, is the statistical analysis and its interpretation appropriate?

Yes

Are all the source data underlying the results available to ensure full reproducibility?

Yes

Is the study design appropriate and is the work technically sound?

Yes

Are the conclusions drawn adequately supported by the results?

Yes

Are sufficient details of methods and analysis provided to allow replication by others?

Yes

Reviewer Expertise:

Neurology (epilepsy), epidemiology, health data science

I confirm that I have read this submission and believe that I have an appropriate level of expertise to confirm that it is of an acceptable scientific standard.

# Reviewer report: Reviewer response for version 2

**DOI:** 10.21956/wellcomeopenres.18112.r95088

**Published:** 2024-09-18

**Authors:** Chryso Pallari

**Affiliations:** 1 University of Nicosia Medical School, Engomi, Cyprus

As a general comment, the work described here is very interesting and the manuscript is well written. 

 However, please consider some minor comments below:

 1)In the abstract, please mention the year in which you are referring to, when you talk about 22nd of May and 24th of May.

 2)In the Methods section, I believe is crucial to mention what regression models were built for the specific analysis. 

 3)Introduction is very well written. However, I would like to read more on the already existing online tools, so the reader can get an idea about why this work is important.

Is the work clearly and accurately presented and does it cite the current literature?

Yes

If applicable, is the statistical analysis and its interpretation appropriate?

Yes

Are all the source data underlying the results available to ensure full reproducibility?

Yes

Is the study design appropriate and is the work technically sound?

Yes

Are the conclusions drawn adequately supported by the results?

Yes

Are sufficient details of methods and analysis provided to allow replication by others?

Partly

Reviewer Expertise:

epidemiology, infectious diseases, COVID-19 pandemic, excess mortality

I confirm that I have read this submission and believe that I have an appropriate level of expertise to confirm that it is of an acceptable scientific standard, however I have significant reservations, as outlined above.

# Reviewer report: Reviewer response for version 2

**DOI:** 10.21956/wellcomeopenres.18112.r41538

**Published:** 2020-11-27

**Authors:** Akshaya Srikanth Bhagavathula

**Affiliations:** 1 Institute of Public Health, College of Medicine and Health Sciences, United Arab Emirates University, Abu Dhabi, United Arab Emirates

I appreciate the authors' revision and they provided satisfactory reply to my comments. In this regard, no further changes are required and I recommend for indexing.

Is the work clearly and accurately presented and does it cite the current literature?

Yes

If applicable, is the statistical analysis and its interpretation appropriate?

Yes

Are all the source data underlying the results available to ensure full reproducibility?

Yes

Is the study design appropriate and is the work technically sound?

Yes

Are the conclusions drawn adequately supported by the results?

Yes

Are sufficient details of methods and analysis provided to allow replication by others?

Yes

Reviewer Expertise:

NA

I confirm that I have read this submission and believe that I have an appropriate level of expertise to confirm that it is of an acceptable scientific standard.

# Response

**Published:** 2020-11-27

**Authors:** Michael Poon

**Affiliations:** Dr, UK

Thank you for your prompt reply and we appreciate your feedback on our manuscript.

# Reviewer report: Reviewer response for version 1

**DOI:** 10.21956/wellcomeopenres.17614.r39786

**Published:** 2020-08-27

**Authors:** Nicola Luigi Bragazzi

**Affiliations:** 1 Laboratory for Industrial and Applied Mathematics (LIAM), Department of Mathematics and Statistics, York University, Toronto, ON, Canada

The authors described trends of excess mortality in the UK stratified by nation and cause of death, and developed an online tool for reporting the most up to date data on excess mortality.

 The article is outstanding, well-conducted and well-reported according to high scientific standards. I suggest the authors elaborate more on the concept of "excess mortality". The authors should also refer to other research and tools, such as the EuroMOMO initiative. The authors could quote and discuss the following papers: Paget *et al.* (2019 ^1^) and Iuliano *et al.* (2018 ^2^).

Is the work clearly and accurately presented and does it cite the current literature?

Partly

If applicable, is the statistical analysis and its interpretation appropriate?

Yes

Are all the source data underlying the results available to ensure full reproducibility?

Yes

Is the study design appropriate and is the work technically sound?

Yes

Are the conclusions drawn adequately supported by the results?

Yes

Are sufficient details of methods and analysis provided to allow replication by others?

Yes

Reviewer Expertise:

Epidemiology, biostatistics, mathematical modeling

I confirm that I have read this submission and believe that I have an appropriate level of expertise to confirm that it is of an acceptable scientific standard, however I have significant reservations, as outlined above.

## References

[1] : Global mortality associated with seasonal influenza epidemics: New burden estimates and predictors from the GLaMOR Project. *J Glob Health* .2019;9(2) : 10.7189/jogh.09.020421 020421 10.7189/jogh.09.020421 31673337 PMC6815659

[2] : Estimates of global seasonal influenza-associated respiratory mortality: a modelling study. *The Lancet* .2018;391(10127) : 10.1016/S0140-6736(17)33293-2 1285-1300 10.1016/S0140-6736(17)33293-2 PMC593524329248255

# Response

**Published:** 2020-11-16

**Authors:** Michael Poon

**Affiliations:** Dr, UK

*Please see our point-by-point response to the reviewer's comments.*

**The authors described trends of excess mortality in the UK stratified by nation and cause of death, and developed an online tool for reporting the most up to date data on excess mortality.**

**The article is outstanding, well-conducted and well-reported according to high scientific standards.**

*Response: Thank you for the positive comments.*

**I suggest the authors elaborate more on the concept of “excess mortality”. The authors should also refer to other research and tools, such as the EuroMOMO initiative. The authors could quote and discuss the following papers: Paget et al. (20191) and Iuliano et al. (20182).**

*Response: We have clarified the discussion and added references to other data visualization tools and the distinctive features of our tools compared to others into the manuscript (under “Strengths and Limitations”, first paragraph). In particular, we have clarified that our intention was not to monitor Covid-19 deaths but rather other underlying causes and non-COVID deaths due to the pandemic. We have discussed the concept of excess mortality and further clarified our definition of excess mortality in our manuscript (under “Definitions”, second paragraph).*

# Reviewer report: Reviewer response for version 1

**DOI:** 10.21956/wellcomeopenres.17614.r39789

**Published:** 2020-07-31

**Authors:** Akshaya Srikanth Bhagavathula

**Affiliations:** 1 Institute of Public Health, College of Medicine and Health Sciences, United Arab Emirates University, Abu Dhabi, United Arab Emirates

Thank you for providing this opportunity to review this interesting manuscript. Although the authors have done interesting work, there are some comments and suggestions that can help to improve the quality of work. 

1. The title is misleading, according to their the authors need to explain the design, implementation, and working process of the TRACKED software, but it is not mentioned in the methods. 
2. It is better to consider the extent of excessive death in two patterns. a) Past five years to before COVID and b) during the COVID period (from the first case of COVID-19 reported in the UK).
3. I am sure there are other applications similar to what the authors mentioned in the world. Its better to list them and describe how the TRACKER software is different and validation of the data is done to quantify the results would be interesting. 
4. It is interesting to discuss death due to respiratory disease are much lower than the expected rate and this is particularly lower in the COVID-19 period. Can the authors explain why this differences occurred?

Is the work clearly and accurately presented and does it cite the current literature?

Yes

If applicable, is the statistical analysis and its interpretation appropriate?

Yes

Are all the source data underlying the results available to ensure full reproducibility?

Yes

Is the study design appropriate and is the work technically sound?

Yes

Are the conclusions drawn adequately supported by the results?

Yes

Are sufficient details of methods and analysis provided to allow replication by others?

Yes

Reviewer Expertise:

Infectious diseases, Global Health, Chronic diseases, Public Health and Gastroenterology.

I confirm that I have read this submission and believe that I have an appropriate level of expertise to confirm that it is of an acceptable scientific standard, however I have significant reservations, as outlined above.

# Response

**Published:** 2020-11-16

**Authors:** Michael Poon

**Affiliations:** Dr, UK

*Please see our point-by-point response to the reviewer's comments.*

**Thank you for providing this opportunity to review this interesting manuscript. Although the authors have done interesting work, there are some comments and suggestions that can help to improve the quality of work.**

**1. The title is misleading, according to their the authors need to explain the design, implementation, and working process of the TRACKED software, but it is not mentioned in the methods.**

*Response: We have added more details about the process of the online tool in the Methods section as requested by the reviewer. This interactive tool was constructed using R Shiny (* *https://shiny.rstudio.com/* *) to build the web application in R. The R script automatically downloads and extracts the latest mortality data from Office of National Statistics, National Records of Scotland, and Northern Ireland Statistics and Research Agency, into an analysable format. Subsequent processes generated the graphs and figures displayed in the web application using the stated R packages. All associated codes are available in* *https://github.com/michaelpoontc/tracked* *. Our revised Methods section (under “Data processing and statistical procedures”, first paragraph) clarifies the implementation and processes of our interactive web application.*

**2. It is better to consider the extent of excessive death in two patterns. a) Past five years to before COVID and b) during the COVID period (from the first case of COVID-19 reported in the UK).**

*Response: Our original intent was to examine the indirect effects of initial lockdown mitigation measures on non-COVID excess deaths and not necessarily COVID directly. As the pandemic has stretched on for many months and is continuing to evolve, A strength of our online tool is that it allows the user to dynamically look at these trends at any time-period of interest (see tabs under “Nations), which will be useful even beyond the COVID pandemic. To illustrate the interactive component of our web application, you may select the time period within the England tab to display mortality data in England between 03 Jan 2020 and 31 Jan 2020 – before the first case of confirmed COVID-19 case. All numbers at the top are specific for this period. Comparing this to the period when excess mortality was highest during the first wave (between 27 March 2020 and 08 May 2020), these numbers are vastly different.* *Excess deaths data is dynamic during the pandemic and our interactive tool allows the user to examine the data depending on temporal time period or death outcome of interest. This manuscript aims to only summarize some of the tool’s capabilities and ability to help researchers to better understand mortality data during the COVID-19 pandemic.*

**3. I am sure there are other applications similar to what the authors mentioned in the world. Its better to list them and describe how the TRACKER software is different and validation of the data is done to quantify the results would be interesting.**

*Response: Yes indeed. Since our manuscript submission, there have been several online sources that display different aspects of COVID-related mortality (* *https://ourworldindata.org/mortality-risk-covid?country=~GBR#case-fatality-rate-of-covid-19-compared-to-other-diseases* *,* *https://www.ft.com/content/a2901ce8-5eb7-4633-b89c-cbdf5b386938* *,* *https://www.kingsfund.org.uk/publications/deaths-covid-19#impact-on-overall-mortality* *,* *https://www.euromomo.eu/graphs-and-maps/* *,* *https://public.tableau.com/profile/phs.covid.19#!/vizhome/COVID-19DailyDashboard_15960160643010/Overview* *). We have now referred to these in our manuscript (under “Strengths and Limitations”, first paragraph). However, our web application is distinct in several aspects. First, this is the only available tool to collate UK-wide data into one platform in which users can explore up-to-date data dynamically. This is important because the four devolved nations of the UK have different public health strategies for the COVID-19 pandemic. Our web application facilitates comparison and view across all four nations. Secondly, non-COVID-related deaths are not always presented in publicly available data or online tools. These non-COVID excess deaths may be attributed to indirect effects of the pandemic, such as public health policies, changes in hospital services, and other mitigation strategies. Lastly, we present cause-specific mortality in Scotland, which is not available in other existing online tools. While this is helpful to interpret the non-COVID-related deaths, we recognise that the numbers are small and comparison between nations is not possible. However, the data adds granularity to non-COVID-related deaths that is otherwise not available elsewhere. We have now added these into our manuscript (under “Strengths and Limitations”, first paragraph). On the point of validity, we use publicly available data published by national statistics agencies. If there were errors in these data that led to the agencies updating them accordingly, this online application will also display those latest, corrected data because of the background workings explained in the methods section (also see https://github.com/michaelpoontc/tracked for codes for further detail if required).*

*4.* **It is interesting to discuss death due to respiratory disease are much lower than the expected rate and this is particularly lower in the COVID-19 period. Can the authors explain why this differences occurred?**

*Response:* *As these are observational data, we cannot know for sure why rates were low. However, there are some likely plausible hypotheses: 1) COVID-related death is a new distinct respiratory cause of death. At present, COVID-related deaths encompass all deaths mentioning COVID-19, which may or may not be the main or contributory cause of death. Many deaths that might otherwise normally be attributed as “respiratory” will be covered by this broad definition, which could explain the paradoxical reduction in respiratory deaths. 2) the UK government reported a relatively low influenza activity in the UK 2019-2020 (https://www.gov.uk/government/statistics/annual-flu-reports). Since seasonal influenza epidemics are an important cause for deaths due to respiratory diseases, this was likely to have attributed to the observation. We have commented on this in our discussion (under “Discussion”, third paragraph).*
